# Intracellular lipid binding protein family diversity from Oyster *Crassostrea gigas*: genomic and structural features of invertebrate lipid transporters

**DOI:** 10.1038/srep46486

**Published:** 2017-04-21

**Authors:** Guilherme de Toledo-Silva, Guilherme Razzera, Flavia Lucena Zacchi, Nestor Cubas Wendt, Jacó Joaquim Mattos, Afonso Celso Dias Bainy

**Affiliations:** 1 Laboratory of Biomarkers of Aquatic Contamination and Immunochemistry - LABCAI, Biochemistry Department, Federal University Santa Catarina, Florianópolis, Brazil; 2 Aquaculture Pathology Research Center - NEPAQ, Federal University of Santa Catarina, Florianópolis, Brazil

## Abstract

Intracellular lipid binding proteins (iLBPs) play a role in the transport and cellular uptake of fatty acids and gene expression regulation. The aim of this work was to characterize the *iLBP* gene family of the Pacific oyster *Crassostrea gigas*, one of the most cultivated marine bivalves in the world, using bioinformatics and molecular biology approaches. A total of 26 different *iLBPs* transcripts were identified in the Pacific oyster genome, including alternative splicing and gene duplication events. The oyster *iLBP* gene family seems to be more expanded than in other invertebrates. Furthermore, 3D structural modeling and molecular docking analysis mapped the main amino acids involved in ligand interactions, and comparisons to available protein structures from vertebrate families revealed new binding cavities. Ten different *CgiLBPs* were analyzed by quantitative PCR in various tissues of *C. gigas,* which suggested differential prevalent gene expression of *CgiLBPs* among tissue groups. The data indicate a wider repertoire of *iLBPs* in labial palps, a food-sorting tissue. The different gene transcription profiles and reported docking systems suggest that the iLBPs are a non-generalist ligand binding protein family with specific functions.

Intracellular lipid-binding proteins (iLBPs) are a group of low molecular mass proteins involved in the intracellular transport of fatty acids and other hydrophobic molecules. The iLBPs are a family of fatty acid (FABP), retinol (CRBP) and retinoic acid (CRABP) binding proteins^1^,^2^,^3^. iLBPs from different organisms usually have 130 amino acids, and have a wide variation in amino acid identity (20 to 70%). However, the tertiary structures of these proteins are highly conserved and particularly consist of a cavity formed by ten anti-parallel strands and two helices that can bind and hold lipophilic compounds such as fatty acids^3^,^4^,^5^.

The iLBPs of vertebrates were classified into four subfamilies according to ligand binding preferences. Subfamily I includes CRBP and CRABP, subfamily II includes FABP1 and FABP6, subfamily III includes FABP2, and subfamily IV includes the most members (FABP3, FABP4, FABP5, FABP7, FABP8, FABP9 and FABP12)^5^,^6^. However, the inclusion of invertebrate iLBPs, which differ from vertebrate iLBPs, slightly changed the relationships among iLBP family members^7^. Regardless of several studies about invertebrate iLBPs^5^,^8^,^9^,^10^, there is scarce information about gene/protein diversities and their 3D structure-function relationships. Despite the recent availability of genomic and transcriptomic public databases, genome-wide surveys of this multigene family in invertebrate species are limited^11^. Currently, it is reasonable to perform such studies to characterize iLBPs in invertebrates by their diversity and genomic organization.

*Crassostrea gigas* is one of the most cultivated bivalves in the world and considered a reference species for molecular studies in mollusks^12^,^13^. The Pacific oyster is a typical sentinel organism for biomonitoring studies and is widely used to evaluate environmental pollutant effects since it can accumulate and tolerate these compounds^14^,^15^,^16^. Previous studies have demonstrated upregulation of *C. gigas FABP intestinal-like* gene (GenBank accession ABU41520) after sewage and pharmaceutical exposures^17^,^18^,^19^,^20^. Despite the genome of *C. gigas* is publicly available, thorough studies regarding *iLBPs* are still lacking^21^.

To evaluate the gene/protein iLBP diversity of the Pacific oyster genome, the present study investigates *iLBP* features as: exon/intron boundaries, phylogenetic relationships and gene transcription patterns in different tissues. Furthermore, we modeled 3D proteins and docked fatty acids to map the functional amino acids of these iLBP members. This study provides the first characterized molecular catalog of iLBP putative proteins of a bivalve species, using publicly available data to promote deeper knowledge of an important gene family through the use of bioinformatics and molecular biology techniques.

## Results and Discussion

### RNA-seq mapping, transcript reconstruction and screening for *iLBP* family members

Data from the *Crassostrea gigas* genome and transcriptome were used to analyze the genomic structure of *iLBPs*. The metrics of the short-read mapping and transcript reconstruction were similar or slightly higher than the original work^21^ (Tables S1 and S2). These differences can be explained by improvements made to the more recent bioinformatics programs.

After sequence annotation, we identified 25 putative *iLBP* sequences among the transcripts (Table S3). Their respective open reading frames (ORFs) were compared to the Pacific oyster entries deposited in the NCBI non-redundant protein databank (nr) (Table S4). Among the sequences of putative proteins, 21 had 100% coverage and identity with annotated sequences in NCBI nr, three were assigned as possible new transcripts derived from alternative splicing, and one was a new hypothetical pseudogene. These results show that the transcript reconstruction procedure was robust, recovering almost all described *C. gigas iLBPs* with the exception of a pseudogene annotated as *CRBP1* (GenBank accession EKC22532.1), whose sequence was directly retrieved from the NCBI repository. This exception could be explained due to the lack of transcription of the analyzed tissues, and could possibly be a pseudogene detected by *ab initio* procedures in the genome sequencing study^21^. By the adopted criteria of gene boundaries (physical localization and common usage of exons, see Methods), Pacific oyster’s *iLBPs* were classified as 14 different genes, ten transcripts variants (synonymous and non-synonymous) and two pseudogenes (Table S3). *C. gigas* presents a wide repertoire of *iLBP* genes compared to the majority of other invertebrates^11^.

### Nomenclature for Pacific oyster iLBPs

There is no official standard for invertebrate iLBP classification. There are implicit difficulties in establishing orthologous relationships among vertebrate and invertebrate iLBPs due to a distinct evolutionary history; most *iLBP* genes emerged after the event of vertebrate/invertebrate split (~600–700 mya) and are derived from several duplications of a unique ancestral lipocalin gene^1^,^5^.

**Figure 1:**
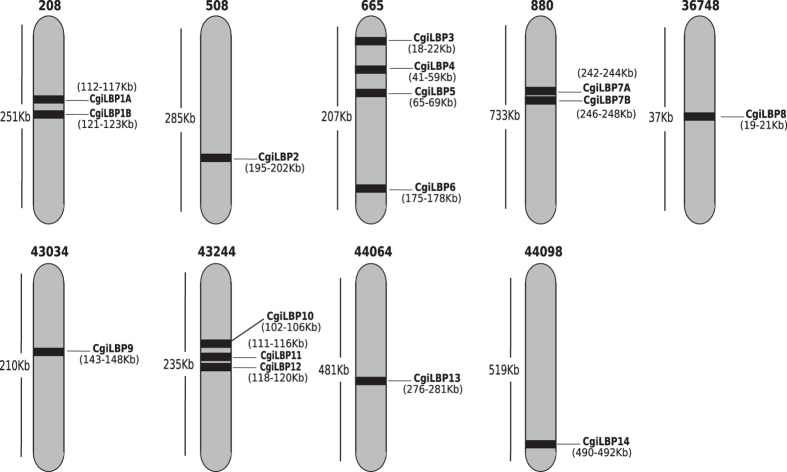
Genomic distribution of *CgiLBPs* genes along different scaffolds from Pacific oyster’s genome assembly (version 9.0). Scaffold sizes, gene length and gene position are not drawn to scale. *CgiLBPs* were named following scaffold order, using letters for gene duplications.

In vertebrates, the initial nomenclature for *FABP* genes was based on the tissue in which it was originally detected (e.g., *fatty acid binding protein, heart-type*). However, the current classification uses numerals after the name (e.g., *FABP1, FABP2*)^22^. In invertebrates, several approaches are found in the literature. A common one uses FABP preceded by the abbreviation for the species name, for example, *EgFABP1* and *EgFABP2* from *Echinoccocus*^23^. An alternative is to adopt the *-like* term after the name of the corresponding putative homolog vertebrate gene, such as the *FABP2-like* gene found in *C. gigas*^17^. However, the automatized functional annotation of recently sequenced genomes and transcriptomes uses homology-based annotation and is responsible for the majority of invertebrate iLBP descriptions in public databases, such as NCBI nr. In general, invertebrate *iLBPs* have more similarity with vertebrate *FABP3 (hearth-like*) genes^5^, creating a bias in automated annotation. Functional characterization of this protein family in invertebrates is a troublesome task, especially in the superphylum Lophotrochozoa, which includes mollusks. Several invertebrate species do not have a well-annotated, publicly available genome to compare and establish reliable orthologous relationships. Thus, a homology-only based annotation of *iLBP* members of mollusks seems to be inappropriate. In a recent study, new *FABPs* were identified in a great number of invertebrate species, and authors arbitrarily named them using a sequential order for the newfound *FABP* genes^11^. Here, a similar approach was used for several *iLBPs* identified after searching the Pacific oyster genome. However, instead of *CgFABPs*, we used *CgiLBPs* as codification of these proteins to prevent misconceptions when talking about functional characterization, since most of them lack experimental and deeper *in silico* characterization. The sequential nomenclature respected the scaffold order, as shown in Fig. 1.

### Phylogeny

**Figure 2:**
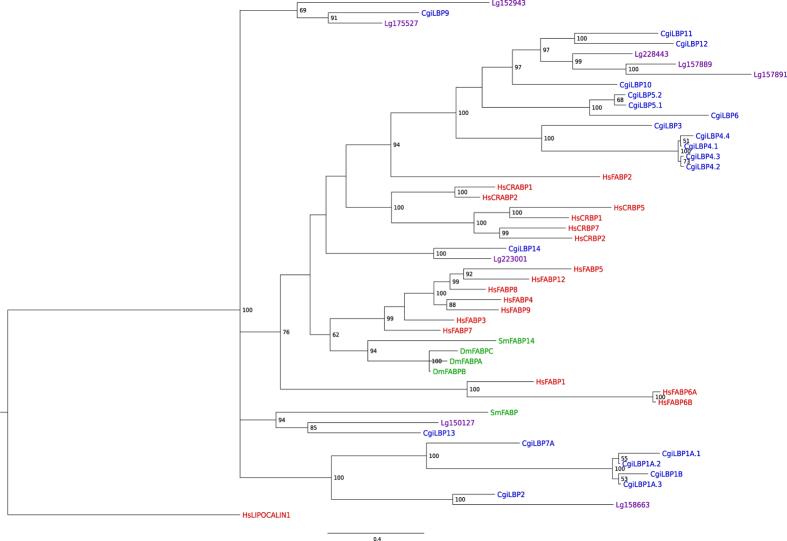
Bayesian tree of *iLBPs* from vertebrate and invertebrate species, inferred from MrBayes v3.2. Tree drawn using FigTree setting *Lipocalin 1* gene from *H. sapiens* as outgroup. Hs stands for *Homo sapiens* (red), Dm for *Drosophila melanogaster* (green), Sm for *Schistosoma mansoni* (green) and Lg for *Lottia gigantea* (purple). *CgiLBPs* (blue) were named according to this study. Posterior probabilities higher than 50 are shown. Vertebrate *iLBP* subfamilies I to IV are depicted in red boxes followed by their respective subfamily number.

We constructed phylogenetic trees for *iLBPs* from vertebrate and invertebrate species. Both Bayesian and maximum likelihood approaches resulted in similar topologies (Fig. 2, Figure S3). In general, phylogenies were well supported for more recent divergence events, but not for deeper nodes. Within the *iLBP* gene family, there is little sequence similarity and few linear motifs^5^, which is difficulty associated with this type of analysis.

First, the phylogenetic model used for *iLBP* genes in vertebrates was maintained in the phylogram, with *iLBPs* from humans forming its respective subfamilies, in accordance with previous inferences^1^. Considering *iLPB* subfamily I in the literature, no CRBP or CRABP was described in bivalves (*C. gigas* and *Lottia gigantea*), and only one CRABP was reported in invertebrates^24^. The results here are similar, with no invertebrate *iLBP* clustering with vertebrate subfamily I. Similarly, no invertebrate *iLBP* is clustered in subfamily II, composed of FABPs that bind and transport cholesterol and bile acids. Subfamily III is an interesting example, as *CgiLBP4* have higher levels of homology via sequence comparison with *FABP2* from vertebrates. In the proposed phylogenetic tree in Fig. 2, there is good support for clustering *CgiLBP4* and *FABP2* from humans, at least in the Bayesian approach (Fig. 2). In addition to *CgiLBP3, CgiLBP4, CgiLBP5* and *CgiLBP6*, all positioned at the same scaffold (Fig. 1), *CgiLBP10, CgiLBP11, CgiLBP12* and some *L. gigantea* representatives are also clustered in vertebrate subfamily III. Data suggests a putative expansion of subfamily III in bivalves comparing to vertebrate species. Subfamily IV of *iLBPs* is mainly observed in superior vertebrates^1^, so only convergent evolution could explain similarities among *iLBPs* from this subfamily in mollusks. *Drosophila melanogaster* and *Schistosoma mansoni iLBPs*, representing invertebrate species other than Lophotrochozoan animals, formed a cluster independent of mollusks and closer to vertebrate subfamily IV. Sm14 was the first FABP described in platyhelminths^25^ and considered a sister group of subfamily IV of vertebrates^7^, as depicted in the Bayesian and ML trees (Fig. 2, Figure S3).

Other bivalve *iLBPs* and one *S. mansoni* putative *FABP* clustered, forming several subgroups other than vertebrates. However, due to low support, it should be further investigated using more invertebrate species in future studies. One subgroup was composed of *CgiLBP1A* and *CgiLBP1B*, the most derived proteins due to total lack of signature motifs or domains (Table S3), and clustered together with *CgiLBP2, CgiLBP7A* and one *iLBP* from *L. gigantea*. The Pacific oyster *iLBP* gene repertoire is apparently wider than those of other studied invertebrate species, which suggests different phylogenetic relations with vertebrate iLBPs and even to Arthropoda and Platyhelminth *FABPs*. Until there are more detailed studies of iLBP evolutionary history in invertebrates, it is not recommended to name bivalve genes using homology with vertebrate FABPs, as several mollusks genes probably derived independently after the vertebrate/invertebrate split at 600–700 mya. More studies focusing on orthologous relationships among invertebrate species could shed light on these questions and yield a more accurate classification.

### Genomic organization

The genomic structures of identified *iLBPs* were similar to the canonical organization of four exons and three introns, with the exception of the two pseudogenes (Table S3). A recent review about invertebrate *FABPs* shows that *FABP* genes usually follow a similar genomic configuration to vertebrates^11^. For example, the mollusk *L. gigantea* has the majority of *FABP* genes structured as the canonical organization. The size of identified CgiLBPs ranged from 131 to 143 aa, very close to the average size for this protein family^11^. Again, the exceptions were the pseudogenes, which lacked one exon and generated translated sequences approximately 100 aa long.

Concerning *Crassostrea gigas iLBP* splicing variants, eight genes showed no evidence of alternative splicing in analyzed tissues. *CgiLBP10* (Figure S1), *CgiLBP11* and *CgiLBP12* (Figure S2) present identical variants at nucleotide level and are closely located at scaffold 43244 (Fig. 1), with *CgiLBP11* and *CgiBLP12* overlapping each other (Figure S2). Identities in protein sequences are approximately 45% between *CgiLBP10* and the other two genes, and approximately 50% between *CgiLBP11* and *CgiLBP12*. These levels of identity are considered above average in *CgiLBPs*, suggesting recent local genomic duplications.

**Figure 3:**
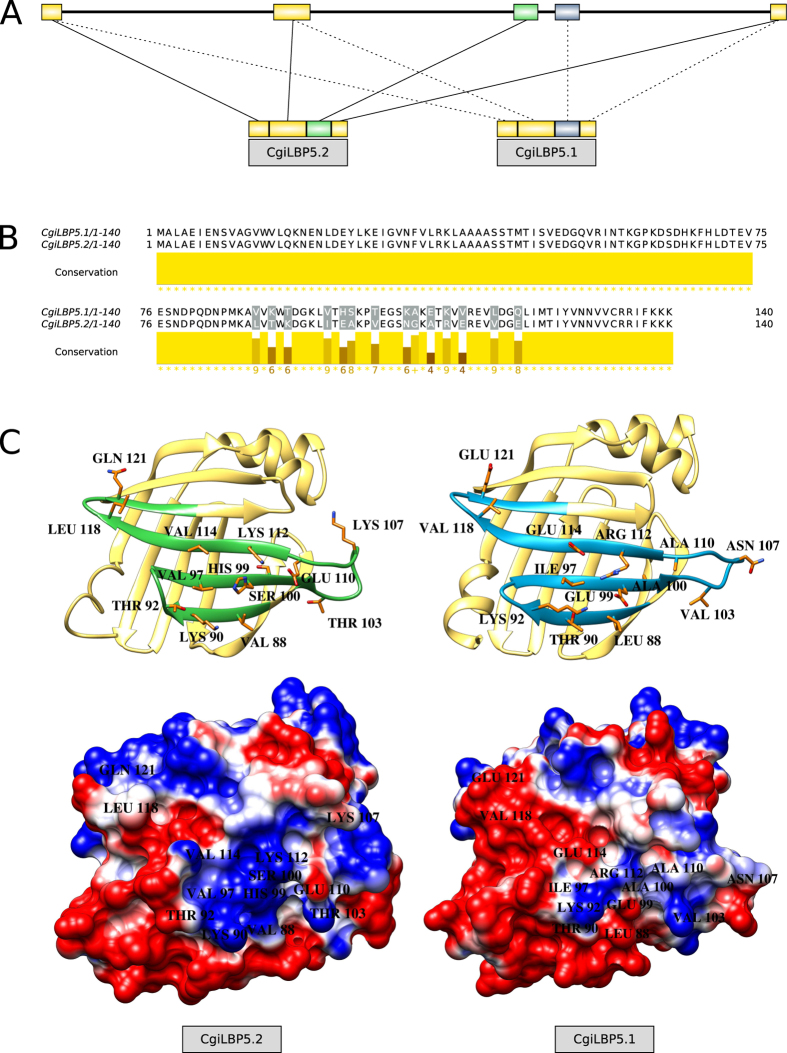
(**A**) *CgiLBP5* genomic structure, depicting the mutually exclusive exon splicing. (**B**) MUSCLE alignment of amino acid sequence of transcripts *CgiLBP5.1* and *CgiLBP5.2*, showing non-synonymous alterations in the mutually exclusive exons. Graphic representation generated in Jalview v2.1. (**C**) 3D structural models show the amino acid differences on the surface of the proteins between transcripts CgiLBP5.1 and CgiLBP5.2, and charged surface plots show the positive patch (in blue) and negative patch (in red) of CgiLBP5.1 and CgiLBP5.2.

**Figure 4:**
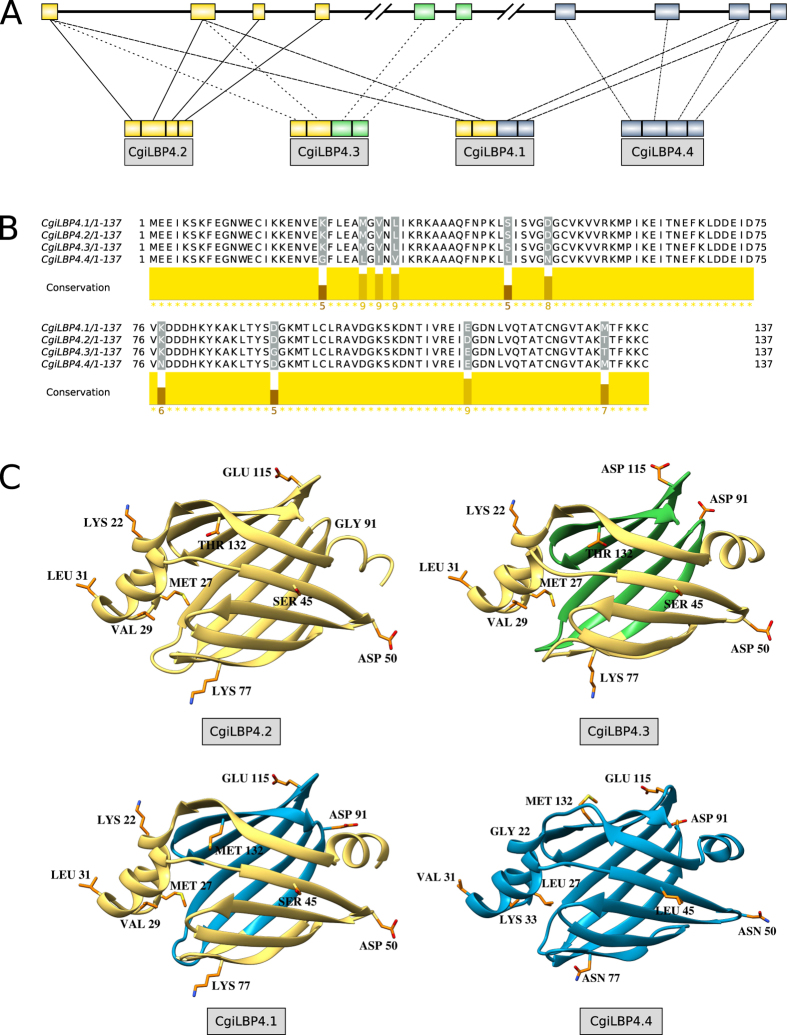
(**A**) *CgiLBP4* genomic structure, depicting the recent duplications and common usage of exons. (**B**) MUSCLE alignment of amino acid sequence of transcripts *CgiLBP4.1, CgiLBP4.2, CgiLBP4.3* and *CgiLBP4.4*. Graphic representation generated in Jalview v2.1. (**C**) 3D structural models showing amino acid substitutions in each isoform. Particularly positions 27 and 132 may tune the lipid binding sites.

Three genes showed splicing variants that resulted in alterations in both nucleotide and amino acid sequences: *CgiLBP1A, CgiLBP4* and *CgiLBP5*. These genes present different paradigms regarding genomic structure and the use of alternative splicing. The *CgiLBP5* gene shows a typical mutually exclusive exon alternative splicing mode (Fig. 3A). The third exon either suffered a small local duplication or is a vestige of whole gene duplication. This gene presents two possible transcripts in this region (Fig. 3B). Another gene that shows patterns of alternative splicing is *CgiLBP4*. This gene appears to be a mix of very recent gene duplication (~90% identity) and common use of exons (Fig. 4A). The variant *CgiLBP4.1* unites distant exons, and this genomic region is characterized as one gene. *CgiLBP4* gene has been considered a biomarker for exposure to contaminants such as domestic sewage and ibuprofen^17^,^18^,^19^,^20^. Named as *FABP2*-*like or FABP2 intestinal type* in such studies, the primer pairs for PCR quantification predicts amplification of both *CgiLBP4.1* and *CgiLBP4.4* putative transcripts. It is not clear whether these proteins are involved in another biological role other than fatty acid transportation. The hypothesis that CgiLBPs participate directly in response to xenobiotic exposure needs to be evaluated; the higher levels of *CgiLBP4* transcripts observed in these studies could be involved mobilizing and transporting lipids to enhance energy production to cope with the metabolic demands during and after stress caused by contaminant exposure. The probable duplication events that occurred in this region and the preservation of copies in the Pacific oyster genome suggest an important role for the *CgiLBP4* gene. Such duplications of stress related genes were significantly retained in the Pacific oyster genome^21^. Studies should explore how all variants respond to contaminant exposure, or the mechanisms involved in their regulation.

**Figure 5:**
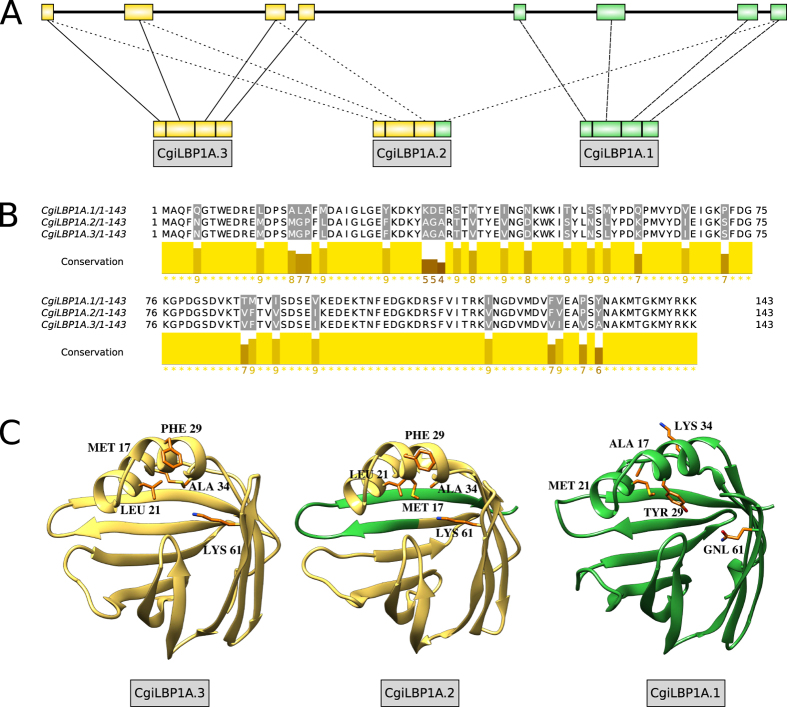
(**A**) *CgiLBP1* genomic structure, depicting the recent duplications and common usage of exons. (**B**) MUSCLE alignment of amino acid sequence of transcripts *CgiLBP1.1, CgiLBP1.2* and *CgiLBP1.3*. Graphic representation generated in Jalview v2.1. (**C**) 3D structural models showing amino acid substitutions inside the lipid binding cavities.

Lastly, we describe *CgiLBP1A* as another example of alternative splicing of *iLBPs* in *Crassostrea gigas* (Fig. 5). Similar to *CgiLBP4* region, it probably underwent gene duplication. *CgiLBP1B* sequence shows 71.76%, 85.50%, 87.02% amino acid identity to *CgiLBP1A.1, CgiLBP1A.2* and *CgiLBP1A.3* transcripts, respectively. *CgiLBP1A* and *CgiLBP1B* genes were both detected at scaffold 208, separated by ~4 Kb. An interesting observation is that these genes lack the typical Lipocalin domain (CL0116) from Pfam libraries, and the three FATTYACIDBP motifs (PR0078) from PRINTS database. The total lack of any domain/motif was exclusive of *CgiLBP1* duplicated genes, as other *CgiLBPs* also failed to detect some of these signature domains or motifs (Table S3). This region probably offers a wide range of *CgiLBP1* functionalities, which would account for functional gene duplicates and the presence of alternative splicing in *CgiLBP1A*.

### Molecular Modeling

Understanding the iLBPs functionalities is a challenging task, considering different binding capacities, multiple ligand binding sites, cavity flexibility and cellular localization^26^,^27^. Two different approaches were used here for 3D structural modeling. The first was a hybrid methodology using threading and homology modeling from the I-Tasser suite, which is able to predict structural features of non-conserved regions by fragment assembly simulations^28^. The second was a homology modeling-only method from the SWISS-MODEL suite^29^, which preserves the similarities from a single template protein structure. SWISS-MODEL was only used for modeling the most conserved protein cavities, required in molecular docking analysis, as discussed below. Despite the low sequence identity among *C. gigas* and mammalian iLBPs, 3D models obtained by threading-homology modeling showed high quality models (TM-score higher then 0.7). All members display a conserved FABP structural fold with 2 α-helices and 10 β-strands, except for CgiLBP1B, which exhibits a shorter N-terminal region, and α-helix 2 was not modeled on the helix-loop-helix motif region (Figure S4). Therefore, CgiLBP1B seems to encode a truncated protein compared to CgiLBP1A. CgiLBP1, CgiLBP2, CgiLBP3, CgiLBP4, CgiLBP5, CgiLBP10, CgiLBP11 and CgiLBP12 each showed an additional N-terminal helix. Several FABP structures deposited in Protein Data Bank (PDB), such as FABP3 (3WVM), FABP5 (4LKP), FABP8 (4BVM) and FABP9 (4A60), present the N-terminal 3.10 helices that are relevant for folding and binding since they are located at the “backdoor” of ligand cavity. The FABP ligand portal entrance, reported in vertebrates, is composed of α-helix 2 and the loops that connect β-strands CD and β-strands EF^30^. Compared to those FABPs, almost all CgiLBPs seem to have the portal entrance.

CgiLBP5 3D models (Fig. 3C) show the amino acid differences at the surface region of its splicing variants. The charged surface was illustrated to show those differences; a negative patch on CgiLBP5.2 represents the substitution of three amino acids with glutamic acid (GLU^99^, GLU^114^ and GLU^121^) when compared to CgiLBP5.1. The iLBP family has been related to many molecular interaction partners, including nuclear receptors for gene expression regulation^27^; therefore, we can speculate that those surface modifications may reflect different biological roles.

The nuclear localization signal (NLS), which is related to lipid delivery to nuclear receptors, was identified in the CgiLBP family. The typical vertebrate NLS involves residues K21, R29, and K30 in CRABP-II, and K21, R30, and R31 in FABP4, all located within the protein helix-loop-helix^26^,^31^. Based on sequence analysis and 3D models, CgiLBP5, CgiLBP6, CgiLBP10, CgiLBP11 and CgiLBP14 have exposed basic residues at these positions and may be involved in nuclear translocation. It is not clear if the basic residue triad is the only feature associated with nuclear lipid delivery. FABP1 and FABP2 were able to modulate PPARα receptor activation^32^ and do not have the complete basic triad residues. Interestingly, the human FABP2 isoform pattern (E/R/K) at helix-loop-helix is also found in *C. gigas* iLBPs and it is exclusively found in CgiLBP4 isoforms. BLAST analysis strongly suggests *CgiLBP4* is a *FABP2* vertebrate homolog and the NLS signature reinforces this correlation, in addition to clustering by phylogenetic inference (Fig. 2).

*CgiLBP4* presents four non-synonymic transcript variants. Figure 4C shows structural models that emphasize the differences between those variants. The differences at positions THR^132^MET inside the cavity, MET^27^LEU on the portal region and VAL^29^LYS may change the binding cavity proprieties. CgiLBP1A also has differences in the amino acids inside the cavity: LEU^21^MET, PHE^29^TYR, LYS^61^GLN and ALA^34^LYS (Fig. 5C). We suggest that iLBPs, particularly the CgiLBP1A sequences, which are the most divergent molecules of the iLBP repertoire, are good targets for experimental structural data collection and biochemical analysis.

### Docking Analysis

To evaluate ligand binding properties of *C. gigas* iLBPs, we used comparative 3D modeling approach. Ligand bound PDB structures were selected as templates for docking analysis and only the CgiLBP structures most similar to PDB templates were selected for analysis (see methods). Palmitic acid, a saturated fatty acid, is found at high concentrations in *C. gigas*^33^ tissues and was chosen as model ligand to identify saturated fatty acid protein transporters such as vertebrate FABP2^26^. All CgiLBPs analyzed were able to bind palmitic acid. Figure S5 shows the main positions in each protein involved in palmitic acid binding.

**Figure 6:**
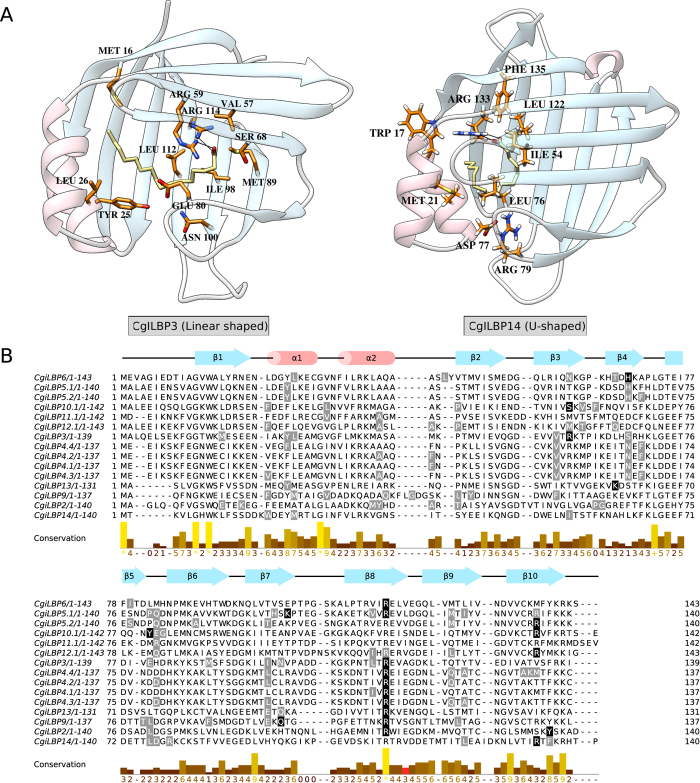
Docking analysis of CgiLBPs using 3D structural models. (**A**) 3D ligand complexes docked with palmitic acid showing the linear shaped (CgiLBP3) and U-shaped (CgiLBP14) tails. (**B**) Sequence alignment of CgiLBPs docked with palmitic acid. Black and gray represent polar and hydrophobic contacts to the ligand, respectively. Secondary structure from CgiLBP 4.1, is depicted schematically above the sequence alignment by pink cylinders (β-strands) and blue arrows (α-helices) representing iLBP fold. The graphical representation was generated in Jalview v2.1.

The key residues involved in ligand head group and hydrophobic interactions were highlighted (Fig. 6B). The conserved ARG residue of β-strand 8 preferentially participates as a hydrogen donor; alternatively, the ARG of β-strand 10 can be substituted as a hydrogen donor. The residues acting as hydrogen donors may determine the fatty acid positioning within the cavity. Typically saturated fatty acids present their linearly shaped tail into the protein cavity, similar to vertebrate FABP2 bound to palmitic acid^26^. In those structures the fatty acid head group is deep inside the cavity and the tail is linear. We found a similar pattern in CgiLBP3, CgiLBP4, CgiLBP5.1, CgiLBP6, CgiLBP9 and CgiLBP13. In these CgiLBP models, the amino acid ARG from β-strand 8 was always the hydrogen-donor to the ligand carboxyl group (Fig. 6A, Figure S5). U-shaped fatty acids positioned in FABP cavities of vertebrates, representative of most of the FABP family, are characterized by a hydrogen bond with the ligand, which involves at least one residue from the pattern ARG/X/TYR of β-strand 10 on C-terminal vertebrate iLBPs^22^. Some CgiLBPs present patterns similar to vertebrate U-shape poses, involving similar binding residues. Those structures are CgiLBP2, CgiLBP5.2, CgiLBP10, CgiLBP12 and CgiLBP14, where we have found ARG or TYR at the C-terminal positions. All those sequences (except CgiLBP2) have ARG in the C-terminal region, which may reflect some preferences for binding (Fig. 6B, Figure S5).

Interestingly, the hydrogen bonding pattern in vertebrate FABP2 involves mainly the ARG from β-strand 8, despite the presence of ARG in the C-terminal region^34^. At least in *Crassostrea gigas*, our data show evidence that the C-terminal ARG does not seem to compete for binding. Instead, when the β-strand 10 pattern is observed, the main hydrogen donor is transferred to the C-terminal part of the protein. This binding mode is usually related to unsaturated fatty acids^26^ and those *C. gigas* proteins may be interesting candidates for the higher demand of PUFAs (poly unsaturated fatty acids) in marine organisms^35^. Our approach was able to highlight the main residues and can be used for mining new sequences with the same pattern in different organisms.

All CgiLBP4s bind palmitic acid similarly to vertebrate FABP2^36^. As shown in CgiLBP4.4 (Fig. 4), the residue MET^132^, which is substituted for THR in CgiLBP4 to interact with ligands, shows evidence of small differences in binding capacities between CgiLBP4 isoforms. Probably, due to recent gene duplication events, the *Crassostrea gigas* genome had an expansion of members involved in the typical saturated fatty acid binding mode from vertebrates, represented by the first binding mode group reported in this work.

The reported bigger cavities identified in FABP1 and FABP6, that may bind cholesterol and derivatives, even two fatty acids in the same cavity^26^,^36^,^37^,^38^,37, were not found in CgiLBP structural models due to lower sequence similarities with those vertebrate members. Concerning phylogenetic analysis, none of the invertebrate *iLBPs* clustered with vertebrate subfamily II, which includes *FABP1* and *FABP*6.

### Gene expression profiles

**Figure 7:**
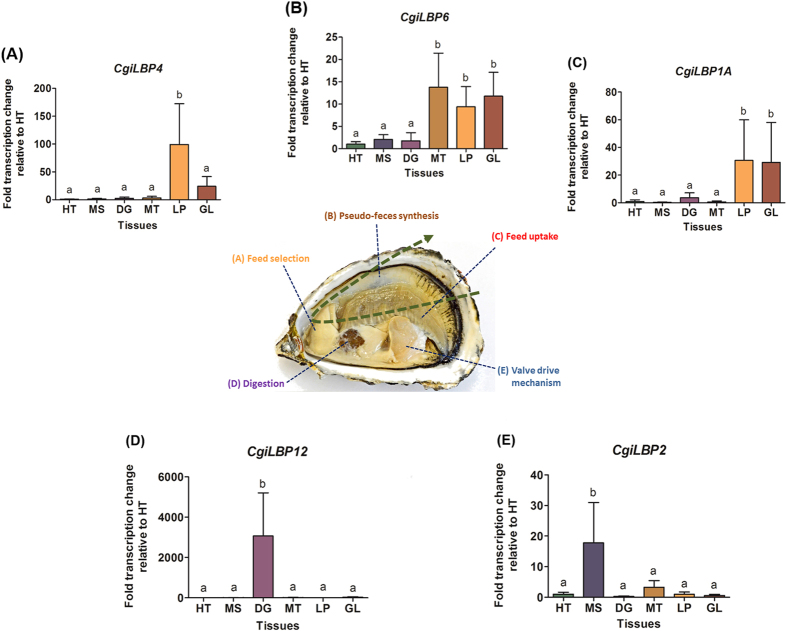
Gene transcription profiles of *Crassostrea gigas iLBP* variants. An example of prevalent variants in each tissue and their corresponding function are presented from panel A to E. The feeding pathway (green dash arrow) is represented by (**A**) labial palps, (**B**) mantle and (**C**) gills. Digestive gland is shown in (**D**) and adductor muscle in (**E**).

*CgiLBPs* transcript levels were evaluated in different tissues of *C. gigas* (Fig. 7). The bivalve feeding process involves several tissues/organs. The filter feeding pathway begins with particle uptake through the gills and transport to the labial palps, which are involved in food selection. The labial palps, in conjunction with the mantle, are also responsible for pseudofeces rejection^38^.

The prevalent transcripts found in gills were *CgiLBP1A, CgiLBP14* and *CgiLBP6*. Oyster gills are directly in contact with the external environment and it is known that the bivalve *Dreissena polymorpha* and *Crassostrea virginica* can uptake lipids directly from water^39^,^40^. Therefore, the function of these *CgiLBP* in gills may be related to lipid uptake from the water column. Other functions for these genes may be related to xenobiotic sensing and transcriptional regulation. The gene products of *CgiLBP1A, CgiLBP14* and *CgiLBP6* may bind lipophilic xenobiotics absorbed by the gills and trigger intracellular signaling cascades leading to transcription of biotransformation genes. *Crassostrea gigas* has been used as a sentinel for aquatic pollution^41^,^42^. High transcript levels of *FABPs,* classified as *CgiLBP4* by the present study, were found in the gills of oysters exposed to sewage^17^,^18^,^20^ and ibuprofen^19^. In this study, *CgiLBP4* was highly expressed in the labial palps. Considering the use of *iLBPs* as biomarkers of aquatic pollution, we suggest investigating *CgiLBP4* in the labial palps.

Remarkably, the labial palps exhibit many differentially expressed *iLBP* members (*CgiLBP1A, CgiLBP6, CgiLBP4, CgiLBP3* and *CgiLBP14*). It is important to note that *CgiLBP1A* and *CgiLBP4* have non-synonymous splice variants, presenting a wider repertoire in this tissue, since iLBP members usually have different ligand binding affinities, which may be relevant for food selection. Considering positive correlation between labial palp size and efficiency on particle selection and its capacity to distinguish between different nutrients, nitrogen/carbon or carbon only sources^43^,^44^, we also suggest a lipid uptake function for this tissue. The labial palps are complex organs in bivalves^38^ and *iLBP* gene expression needs to be evaluated with different tissue segments and closely related species taken into consideration.

In addition to pseudofeces rejection, mantle tissue is associated with energy storage, shell formation and gametogenesis^45^,^46^,^47^. These functions may involve CgiLBP3 and CgiLBP6 proteins since the transcript levels of these genes were significantly higher in this tissue. *CgiLBP12, CgiLBP1B* and *CgiLBP13* transcript rates were 3,064-fold, 629-fold and 21.09-fold, respectively, higher in digestive gland compared to the other tissues, showing 3,064-fold, 629-fold and 21.09-fold respectively. These isoforms may be related to a high energy metabolism and lipid storage^48^.

*CgiLBP9* and *CgiLBP2* were highly expressed in adductor muscle. In bivalves, the main function of this tissue in bivalves is to control the closure of the shells, keep the valves tightly closed for a long time, and make constant, slow valve movements^49^. It is known that bivalve muscle tissue contains limited amounts of stored substrate to generate sufficient energy for these movements, generally sufficient to support contractions for up to three minutes under aerobic conditions and up to 30 seconds under anaerobic conditions. The transcript levels of these *iLBPs* in adductor muscle of *C. gigas* may be related to energy metabolism to maintain the valve movements. In insects, FABP from muscle tissues are also involved in energy metabolism to maintain flight^4^.

No prevalence was found in heart and no difference was found between tissues for *CgiLBP11* (Figure S6). In addition to higher sequence identities (~80%) between the *CgiLBP1* group, distinct patterns were revealed when *CgiLBP1A* and *CgiLBP1B* expression profiles were analyzed*. CgiLBP1A* was prevalent in the gills, as opposed to *CgiLBP1B*, which had higher transcript levels in the digestive gland and encodes a truncated protein. One of the many ways that organisms preserve gene duplications is through subfunctionalization, which leads to tissue specialization regarding gene expression profiles^50^ in many cases. Protein 3D modeling also showed many differences between these isoforms and suggests different functions in respective organs.

### Concluding Remarks

*Crassostrea gigas* presents a wide variety of iLBP proteins, resulting from a process of several duplications and some alternative splicing mechanisms. We reinforce the need for more experimental studies focusing on functional and structural research, as the Pacific oyster’s iLBPs show a distinct evolutionary history when compared to vertebrate’s iLBPs, especially regarding the lack of representatives from classical subfamilies. In addition, CgiLBP1A and CgiLBP1B divergence and the loss of detectable domains suggest a possible new class of iLBPs derived from FABP, and deserves further attention, as qPCR assays demonstrated different gene transcription profiles in some tissues. In light of these observations, we hope that our study will initiate further discussions about iLBPs from Lophotrochozoa species and that a consensus regarding iLBP evolution and functionalities will be reached shortly to benefit both iLBP biology and taxonomy.

## Methods

### Genome screening for iLBP family members

Pacific oyster’s genome assembly (version 9.0) and transcriptome data from RNA-Seq of five different tissues (the gills, digestive gland, labial palps, mantle and adductor muscle) were retrieved from GigaDB (gigadb.org/dataset/100030). Paired-end reads from each issue were separately mapped into available genomic scaffolds using splice-aware aligner TopHat2 v2.1.0^51^ with Bowtie2 mapper v2.2.4^52^, and the parameter --mate-inner-dist was set to 200. Cufflinks v2.2.1^53^ reconstructed the transcripts from each mapping file and Cuffmerge v2.2.1^53^ joined the resulting GTF files into a single unified transcript catalog. Members of the iLBP family were identified by comparison to NCBI’s non-redundant (nr) proteins, Pfam-A v29.0^54^ and PRINTS v42.0^55^ databanks. To compare with nr, blastx option from BLAST + v2.2.30^56^ was used with an e-value filter of 1e-05. PRINTS motifs were searched online (bioinf.manchester.ac.uk/cgi-bin/dbbrowser/fingerPRINTScan/FPScan_fam.cgi) using default parameters. Pfam domains were retrieved using HMMSCAN v3.1b2^57^ with 1e-03 as e-value threshold. Nomenclature of *iLBPs* adopted in this study for transcripts and genes followed scaffold order, without any functional or evolutionary aspects. Duplications were considered at >70% identity between two genes, and named with letters afterwards.

### Pacific oyster iLBP family genomic organization

iLBPs were initially described using transcript and gene information from Cufflinks^53^ as an initial template of *Crassostrea gigas iLBPs*’ genomic structure. Putative *iLBP* homologs previously selected were manually curated using the GTF file generated from Cuffmerge^53^ and genomic scaffolds of *C. gigas* in the Integrated Genome Viewer v2.3 (IGV)^58^ to determine their genetic structures and allow correct grouping of transcripts into genes. In this study, the presence of transcription in the same genomic region (physical location in scaffolds and common usage of exons) was considered the main criteria for establishing an *iLBP* gene. Transcripts were translated and had their most probable open reading frames (ORFs) manually extracted and verified using Expasy translate tool (web.expasy.org/translate/). Amino acid sequences of *iLBPs* were first aligned against the other members transcribed in the same genomic region (therefore the same gene as our established criteria) using MUSCLE^59^ to filter alternative transcripts showing synonymous and non-synonymous differences.

### Phylogeny

Protein datasets from *Homo sapiens, Drosophila melanogaster, Schistosoma mansoni* and *Lottia gigantea* were retrieved from NCBI GenBank. HMMSCAN v3.1b2^57^ and Pfam-A v29.0^54^ were used to scan for Lipocalin domains, in the same manner as *C. gigas iLBPs* were identified. A reciprocal best BLAST hit procedure^60^ was used to search for putative orthologues among the species to complement the datasets.

Amino acid sequences were aligned using MUSCLE^59^. Human Lipocalin 1 (GenBank access NP_002288.1) was selected as an outgroup. The resulting alignment was imported into TOPALi v2.5^61^ and then submitted to Model Selection tool (MrBayes and PhyML), to determine the best substitution model, using BIC (Bayesian information criterion) values to select the models that best fit the data. Phylogenetic trees were generated by PhyML approach through TOPALi v2.5^61^ with 1000 bootstraps, and by MrBayes v3.2^62^ with two runs of 10,000,000 generations, sample rate of 1000, burn-in of 25%. Both procedures used WAG + G as substitution model. Trees were drawn using FigTree v1.4.2 (tree.bio.ed.ac.uk/software/figtree/).

### 3D Modeling and Molecular Docking

The 3D models were built using I-Tasser^28^ suite and SWISS-MODEL^29^. I-Tasser was used with default parameters as a threading assembly approach for protein fold characterization. Models with TM-score higher than 0.5 were selected for analysis. SWISS-MODEL was used on alignment mode to build models for molecular docking. Target *iLBP* sequences were first submitted to blastp analysis against the SWISS-PROT databank and the best hits (10 different sequences) were used for alignments using Clustal omega v1.2.1^63^. The best hit against PDB with ligand and from a non-NMR structure was selected for molecular modeling. Quality of PDB structural models was checked by using Global Model Quality Estimation score (QMQE). iLBP structural modes with QMQE score higher than 0.6 and with sequence identities against the template PDB structure higher than 30% were used for analysis. SWISS-DOCK software^64^ was used for molecular docking. The iLBP models were prepared for docking using Chimera v10.1^65^ at default parameters and AMBER force field^66^. Palmitate ligand was selected from the Zinc database^67^. An accurate mode and 3 Å sidechain flexibility was used for running dock analysis. The lower full fitness pose inside iLBP cavity was selected for ligand binding analysis with LigPlot v4.5.3 software^68^.

### qPCR analysis

*iLBP* transcript levels were evaluated using quantitative PCR (qPCR). To characterize the expression of a unique genomic region, some genes with one or more alternative transcripts were analyzed using primers for the most common exon. Oligonucleotide primers for qPCR were designed using Primer Quest software available at www.idtdna.com (IDT). Selected genes and their respective primer pairs are shown in Table S5.

Oyster (*C. gigas*) samples of heart (HT), adductor muscle (MS), digestive gland (DG), mantle (MT), gills (GL) and labial palps (LP) were collected and frozen in liquid nitrogen. Total RNA from tissues (*n* = 10) was isolated using Qiazol reagent (Qiagen) following the supplier’s protocol with minor modifications. Briefly, 100 mg of each sample was mechanically disrupted in 1 mL Qiazol using a homogenizer (Tissue-Tearor, BioSpec Products). For heart samples, pools of three animals were made to obtain 100 mg of tissue. To check RNA concentration and purity, samples were measured using a NanoDrop ND-1000 Spectrophotometer (Thermo Scientific, Wilmington, DE, USA). 1 μg of total RNA per sample was reverse transcribed using a QuantiTect Reverse Transcription kit (Qiagen).

The quantitative PCR (qPCR) reactions were performed with Quantifast SYBER Green kit (Qiagen) in a Rotor-Gene TM 6000 thermocycler (Qiagen), according to the manufacturer’s instructions. qPCR efficiency (*E*) was determined for each primer pair and checked by running a cDNA calibration curve. Samples were normalized by *Ribosomal_60* *s* gene, chosen by the 2^−Cq^ method^69^. The 2^−ΔCq^ method was applied to the other genes. All data were calibrated by heart group. Statistical analysis was performed using Grubb’s test to detect outliers, and data normality and homoscedasticity were tested by D’Agostino & Pearson and Levene’s test, respectively. When necessary, data were submitted to logarithmic transformation. One-way ANOVA analysis of variance followed by Tukey’s Multiple Comparison’s test was used to compare transcript levels between tissues. Statistics were calculated using Statistica 7 and GraphPad Prism v5.0 software. Differences were considered statistically significant for *p* < 0.05.

## Additional Information

**How to cite this article**: de Toledo-Silva, G. *et al*. Intracellular lipid binding protein family diversity from Oyster *Crassostrea gigas*: genomic and structural features of invertebrate lipid transporters. *Sci. Rep.* **7**, 46486; doi: 10.1038/srep46486 (2017).

**Publisher's note:** Springer Nature remains neutral with regard to jurisdictional claims in published maps and institutional affiliations.

## Supplementary Material

Supplementary Information
