# Supplementary material for: Intracellular lipid binding protein family diversity from Oyster Crassostrea gigas: genomic and structural features of invertebrate lipid transporters
Source: Sci Rep. 2017 Apr 21;7:46486. doi: 10.1038/srep46486 (PMC5399370; doi:10.1038/srep46486)
Supplement: Supplementary Information [file srep46486-s1.doc]

**Intracellular lipid binding protein family diversity from oyster *Crassostrea gigas*: genomic and structural features of invertebrate lipid transporters**

Guilherme de Toledo-Silva^1,+^, Guilherme Razzera^1,+,*^, Flavia Lucena Zacchi^1^, Nestor Cubas Wendt^1^, Jacó Joaquim Mattos^1,2^, Afonso Celso Dias Bainy^1,*^

^1^Laboratory of Biomarkers of Aquatic Contamination and Immunochemistry - LABCAI, Biochemistry Department, Federal University Santa Catarina, Florianópolis, Brazil.

^2^Aquaculture Pathology Research Center - NEPAQ, Federal University of Santa Catarina, Florianópolis, Brazil.

^*^ Corresponding authors: guilherme.razzera@ufsc.br; afonso.bainy@ufsc.br

^+^ These authors contributed equally to this work

**Supplementary figures and tables**

**Table S1.** Results of RNA-Seq short-read mapping.

| **Tissue** | **Input** | **Alignment rate** |
| --- | --- | --- |
| Digestive gland | 37,195,702 | 64.9% |
| Gills | 39,130,698 | 63.9% |
| Mantle | 52,350,722 | 66.9% |
| Labial palps | 33,757,114 | 67.8% |
| Adductor muscle | 38,899,044 | 74.3% |

**Table S2.** Results of transcript reconstruction from tissues of Pacific Oyster.

| **Tissue** | **Genes** | **Isoforms** | **Total transcripts** |
| --- | --- | --- | --- |
| Digestive gland | 45,682 | 6,085 | 51,767 |
| Gills | 46,457 | 5,916 | 52,373 |
| Labial palps | 42,143 | 5,555 | 47,698 |
| Mantle | 50,706 | 6,616 | 57,322 |
| Adductor muscle | 33,093 | 3,757 | 36,850 |
| Merged transcripts | 50,691 | 15,840 | 66,531 |

**Table S3.** Identified *iLBP* transcripts in *Crassostrea gigas* genome. Significant matches against PFAM and PRINTS databases are shown, considering Lipocalin (CL0116) domain for the first and the three FATTYACIDBP motifs (PR00178) for the last. Matches were considered as significant using an e-value threshold of 1e-03.

| **iLBP transcript** | **Length (aa)** | **Exons:Introns** | **PFAM domain** | **N. of PRINTS motifs** |
| --- | --- | --- | --- | --- |
| *Without variants* | | | | |
| *CgiLBP1B* | 132 | 4:3 | No match | No match |
| *CgiLBP2* | 140 | 4:3 | No match | 3 |
| *CgiLBP3* | 139 | 4:3 | Lipocalin (CL0116) | 3 |
| *CgiLBP6* | 143 | 4:3 | Lipocalin (CL0116) | 2 |
| *CgiLBP7A* | 133 | 4:3 | No match | 1 |
| *CgiLBP9* | 137 | 4:3 | Lipocalin (CL0116) | 3 |
| *CgiLBP13* | 131 | 4:3 | Lipocalin (CL0116) | 3 |
| *CgiLBP14* | 140 | 4:3 | Lipocalin (CL0116) | 3 |
| *Synonymous variants* | | | | |
| *CgiLBP10.1* | 142 | 4:3 | Lipocalin (CL0116) | 2 |
| *CgiLBP10.2* | 142 | 4:3 | Lipocalin (CL0116) | 2 |
| *CgiLBP10.3* | 142 | 4:3 | Lipocalin (CL0116) | 2 |
| *CgiLBP11.1* | 142 | 4:3 | Lipocalin (CL0116) | 3 |
| *CgiLBP11.2* | 142 | 4:3 | Lipocalin (CL0116) | 3 |
| *CgiLBP12.1* | 143 | 4:3 | Lipocalin (CL0116) | 2 |
| *CgiLBP12.2* | 143 | 4:3 | Lipocalin (CL0116) | 2 |
| *Non-synonymous variants* | | | | |
| *CgiLBP1A.1* | 143 | 4:3 | No match | No match |
| *CgiLBP1A.2* | 143 | 4:3 | No match | No match |
| *CgiLBP1A.3* | 143 | 4:3 | No match | No match |
| *CgiLBP4.1* | 141 | 4:3 | Lipocalin (CL0116) | 3 |
| *CgiLBP4.2* | 141 | 4:3 | Lipocalin (CL0116) | 3 |
| *CgiLBP4.3* | 141 | 4:3 | Lipocalin (CL0116) | 3 |
| *CgiLBP4.4* | 141 | 4:3 | Lipocalin (CL0116) | 3 |
| *CgiLBP5.1* | 140 | 4:3 | Lipocalin (CL0116) | 2 |
| *CgiLBP5.2* | 140 | 4:3 | Lipocalin (CL0116) | 2 |
| *Pseudogenes* | | | | |
| *CgiLBP7B* | 92 | 3:2 | X | X |
| *CgiLBP8* | 86 | 3:2 | X | X |

**Table S4.** Results of BLASTP analysis of *iLBP* transcripts against NCBI nr.

| **Transcript** | **Description** | **Accession** | **Identity** |
| --- | --- | --- | --- |
| *CgiLBP1A.1* | hypothetical protein CGI_10010025 | EKC40029.1 | 100% |
| *CgiLBP1A.2* | fatty acid-binding protein homolog 6-like isoform X1** | XP_011415437.1 | 97% |
| *CgiLBP1A.3* | fatty acid-binding protein homolog 6-like isoform X1 | XP_011415437.1 | 100% |
| CgiLBP1B | uncharacterized protein LOC105319563 | XP_011415458.1 | 100% |
| CgiLBP9 | fatty acid-binding protein, heart-like | XP_011433631.1 | 100% |
| CgiLBP10.1 | cellular retinoic acid-binding protein 2-like isoform X1 | XP_011426731.1 | 100% |
| CgiLBP10.2 | cellular retinoic acid-binding protein 2-like isoform X1* | XP_011426731.1 | 100% |
| *CgiLBP10.3* | cellular retinoic acid-binding protein 2-like isoform X1* | XP_011426731.1 | 100% |
| *CgiLBP11.1* | fatty acid-binding protein, adipocyte-like isoform X1 | XP_011426734.1 | 100% |
| *CgiLBP11.2* | fatty acid-binding protein, adipocyte-like isoform X1* | XP_011426734.1 | 100% |
| *CgiLBP12.1* | fatty acid-binding protein homolog 6-like | XP_011426736.1 | 100% |
| *CgiLBP12.2* | fatty acid-binding protein homolog 6-like* | XP_011426736.1 | 100% |
| *CgiLBP13* | fatty acid-binding protein, adipocyte-like | XP_011414186.1 | 100% |
| *CgiLBP14* | fatty acid-binding protein homolog 5-like | XP_011417633.1 | 100% |
| *CgiLBP2* | fatty acid-binding protein, brain-like | XP_011412579.1 | 100% |
| *CgiLBP3* | fatty acid-binding protein, heart-like | XP_011446259.1 | 100% |
| *CgiLBP4.1* | fatty acid-binding protein, intestinal-like** | XP_011446262.1 | 99% |
| *CgiLBP4.2* | fatty acid-binding protein, intestinal-like | XP_011446262.1 | 100% |
| *CgiLBP4.3* | fatty acid-binding protein, intestinal-like** | XP_011446262.1 | 98% |
| *CgiLBP4.4* | fatty acid-binding protein, intestinal-like | XP_011446260.1 | 100% |
| *CgiLBP5.1* | fatty acid-binding protein, heart-like isoform X2 | XP_011446264.1 | 100% |
| *CgiLBP5.2* | fatty acid-binding protein, heart-like isoform X1 | XP_011446263.1 | 100% |
| *CgiLBP6* | fatty acid-binding protein, heart-like | XP_011446285.1 | 100% |
| *CgiLBP7A* | fatty acid-binding protein homolog 5-like | XP_011448183.1 | 100% |
| *CgiLBP7B* | fatty acid-binding protein homolog 5-like | XP_011448183.1 | 90% |

* Protein sequence is synonymous ** New alternative transcript

**Table S5.** Primer pair sequences for *CgiLBP* quantitative PCRs.

| **Gene** | **Primer sequence 5' - 3'** | **Amplicon size (bp)** |
| --- | --- | --- |
| *CgiLBP14* | CTTCTTGTAGCAGGGAAACAGTACATC | 127 |
|  | GGTCAAACGTGTCTTGTCATCGAATAA |  |
| *CgiLBP4* | GTGTGTAAAGGTGGTCAGGAAGATG | 101 |
|  | CCTTGTATTTGTGGTCGTCGTCTTT |  |
| *CgiLBP13* | GAAGAAATGACCGAGACGCAGAAG | 118 |
|  | ATTTGATGACAGCAGAAACTCCATTGAT |  |
| *CgiLBP1A* | GTCACGTAACTCATGGCGACTT | 97 |
|  | GCTCCGTCCTACAATGCAAAGA |  |
| *CgiLBP1B* | TGTCAGGGTACAAGCTGGTCAAATAA | 78 |
|  | GTGCAAGAACAACGGTGACCTAC |  |
| *CgiLBP12* | CTACAAGAAGTGGGTGTGGGATTA | 188 |
|  | GCCTTCATTAGAGTTCCTTGCATTTC |  |
| *CgiLBP9* | TGGATGGTGATACCCTCGTAGAA | 110 |
|  | TCCATTGCCAGCAGTCAAGA |  |
| *CgiLBP3* | GTACAAGTCTACGATGTCCTTCAGTG | 140 |
|  | CAATATCCTCGACGGTGTATGTCTG |  |
| *CgiLBP6* | GAGAATCTGGACGGATACCTGAAG | 147 |
|  | GTCTGTGTGTTTGGGACCTTTG |  |
| *CgiLBP2* | TCTCCAATGAAGAGTCTGGTCAATTT | 108 |
|  | CCCTTCTATCCACCGTGTTATGT |  |
| **Normalizing Gene** | **Primer sequence 5' - 3'** | **Amplicon size (bp)** |
| *CgRib60s* | CATTCAAGCGTTCGGGCAATTTA | 121 |
|  | TTGGTTACAGGTCCATTCCATCTT |  |


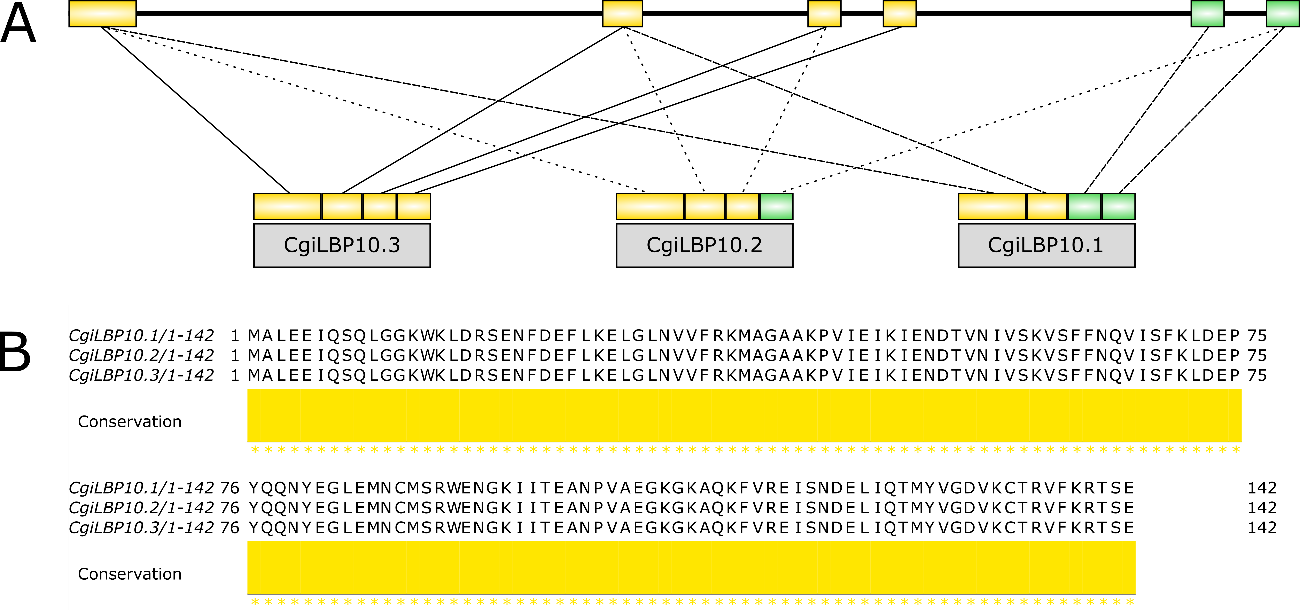


**Figure S1.** (A) Genomic structure of *CgiLBP10* transcripts (B) MUSCLE alignment of *CgiLBP10* amino acid sequences, visualized in Jalview 2.1.

**
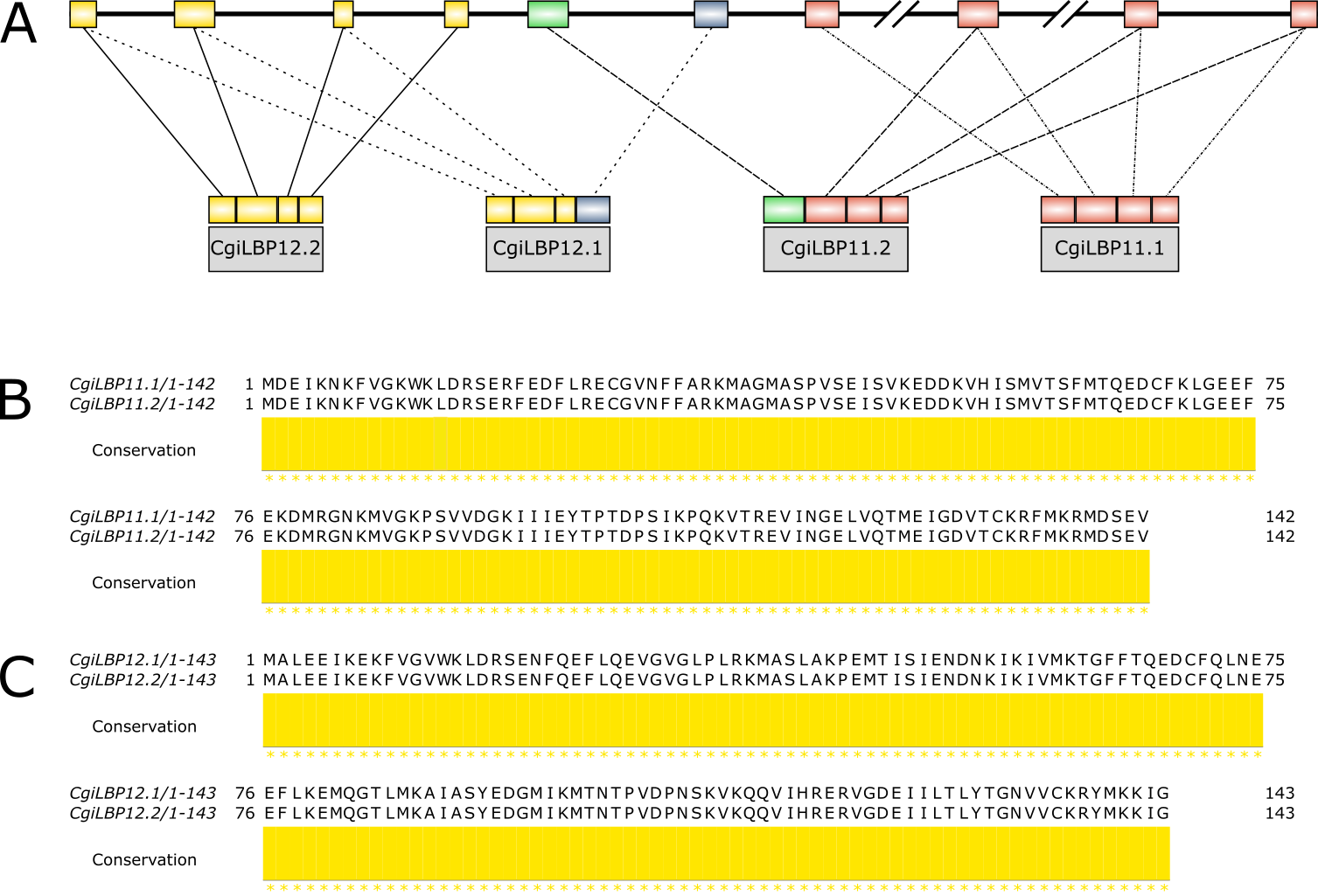
**

**Figure S2.** (A) Genomic structure of *CgiLBP11* and *CgiLBP12* transcripts (B) MUSCLE alignment of *CgiLBP11* amino acid sequences. (C) MUSCLE alignment of *CgiLBP12* amino acid sequences. All alignments visualized in Jalview 2.1.


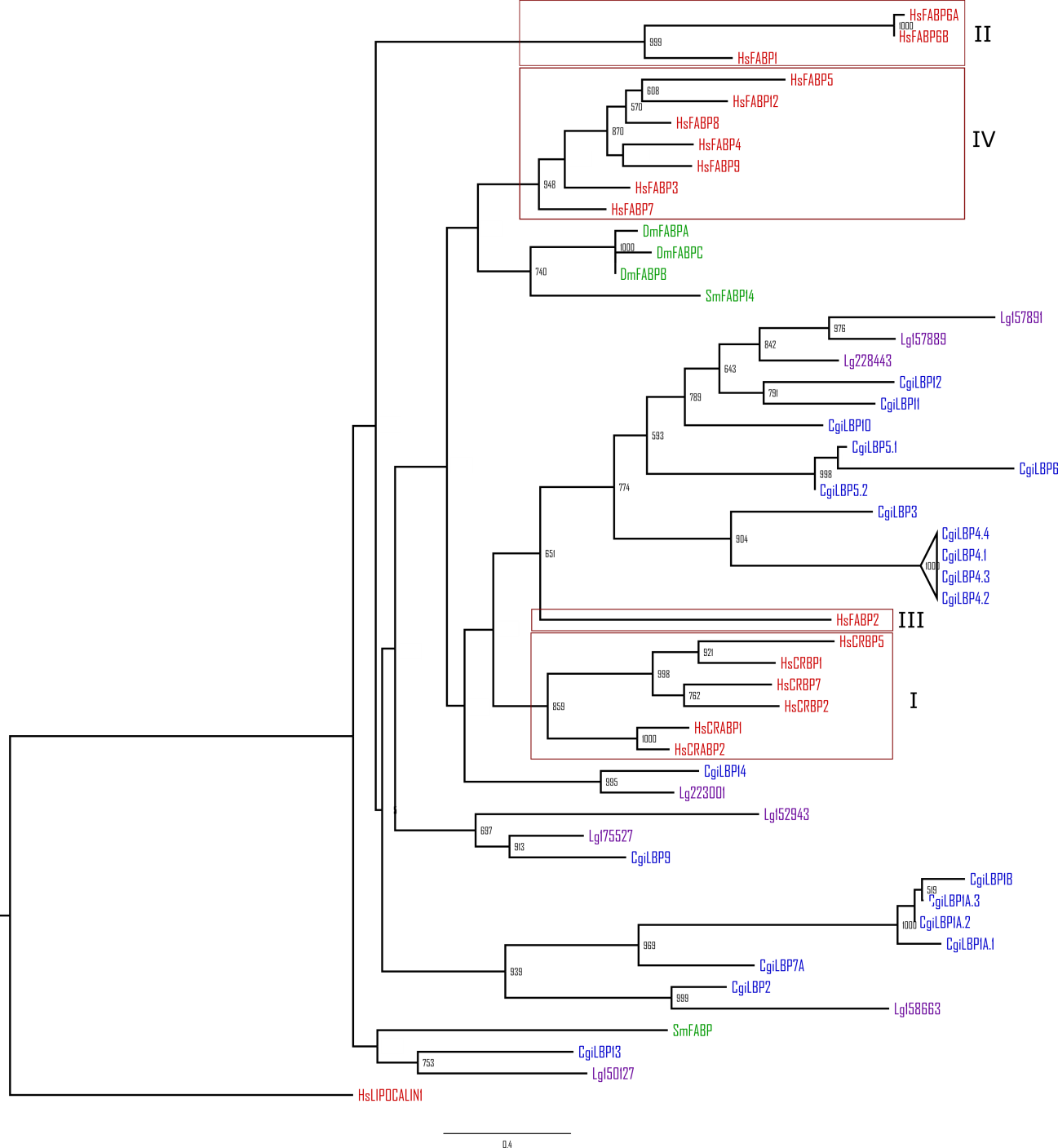


**Figure S3.** ML tree of *iLBPs* from vertebrate and invertebrate species, inferred from PhyML using TOPALI 2.5. Tree drawn using FigTree setting *Lipocalin 1* gene from *H. sapiens* as an outgroup. Hs stands for *Homo sapiens* (red), Dm for *Drosophila melanogaster* (green), Sm for *Schistosoma mansoni* (green) and Lg for *Lottia gigantea* (purple). *CgiLBPs* (blue) were named according to this study. Bootstrap values higher than 500 are shown. Vertebrate *iLBP* subfamilies I to IV are depicted in red boxes followed by their respective subfamily number.


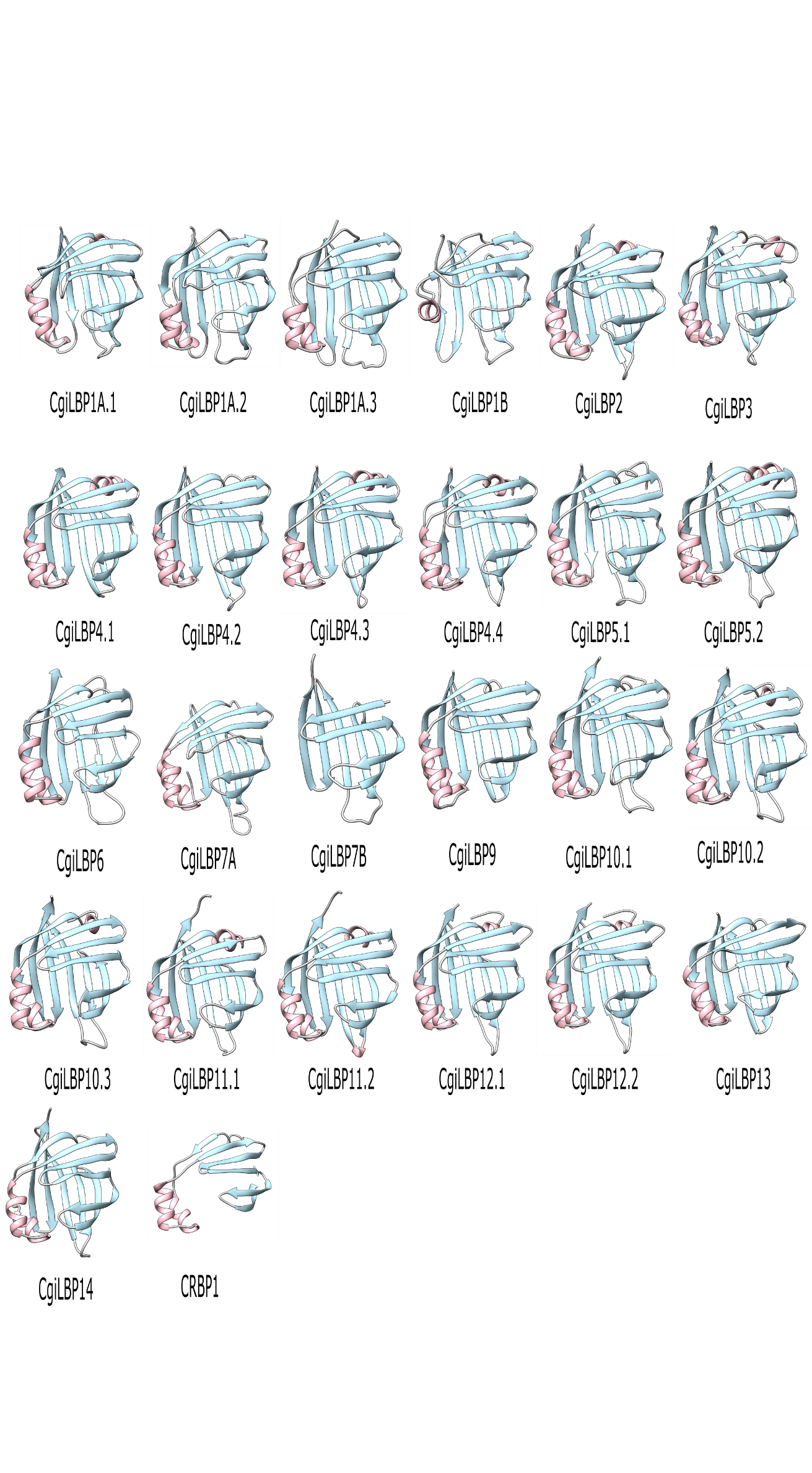


**Figure S4.** 3D structural models presenting the overall *Crassostrea gigas* iLBP foldings. Helices are presented in pink and β-strands in blue. CgiLBP1B, CgiLBP7B and CRBP1 present truncated iLBP folds.

**
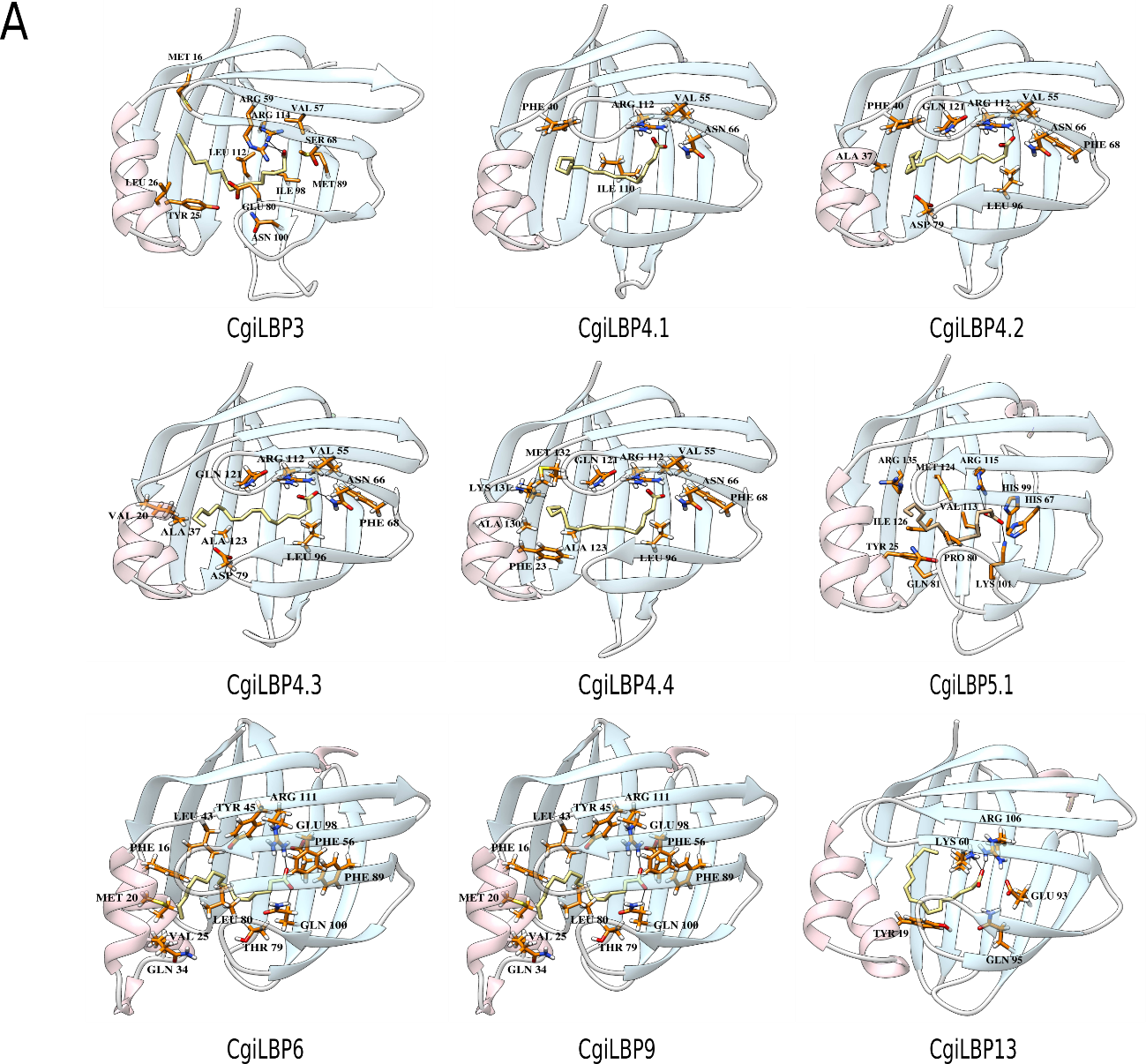
**

**Figure S5.** *Crassostrea gigas* iLBP binding modes. **(A**) the amino acid ARG from β-strand 8 as a hydrogen-donor to palmitic acid (yellow). **(B)** the amino acid ARG from β-strand 10 as a hydrogen-donor to the ligand head group, except CgiLBP2 that uses TYR136. In orange, the key residues involved in ligand binding.

**
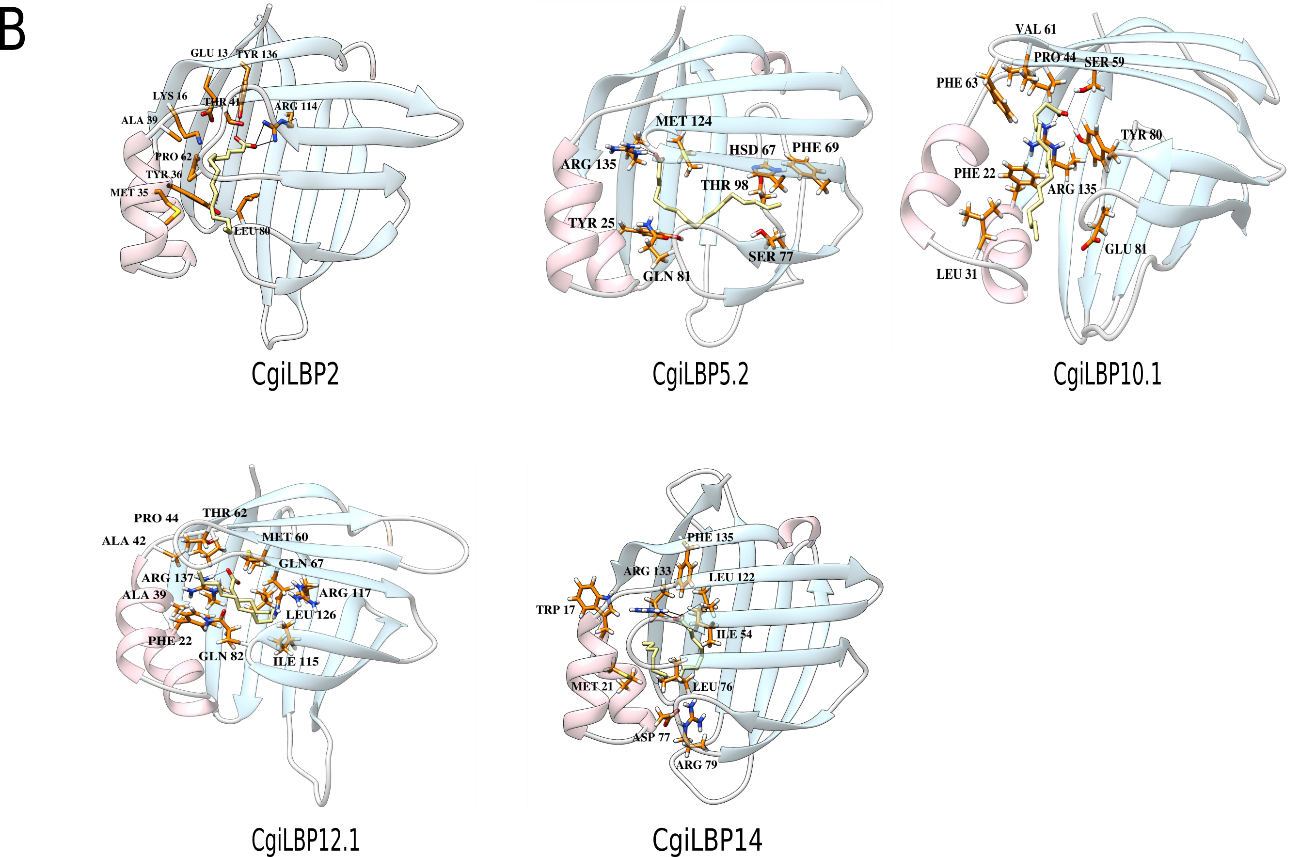
**

**Figure S5.** *Cont.*


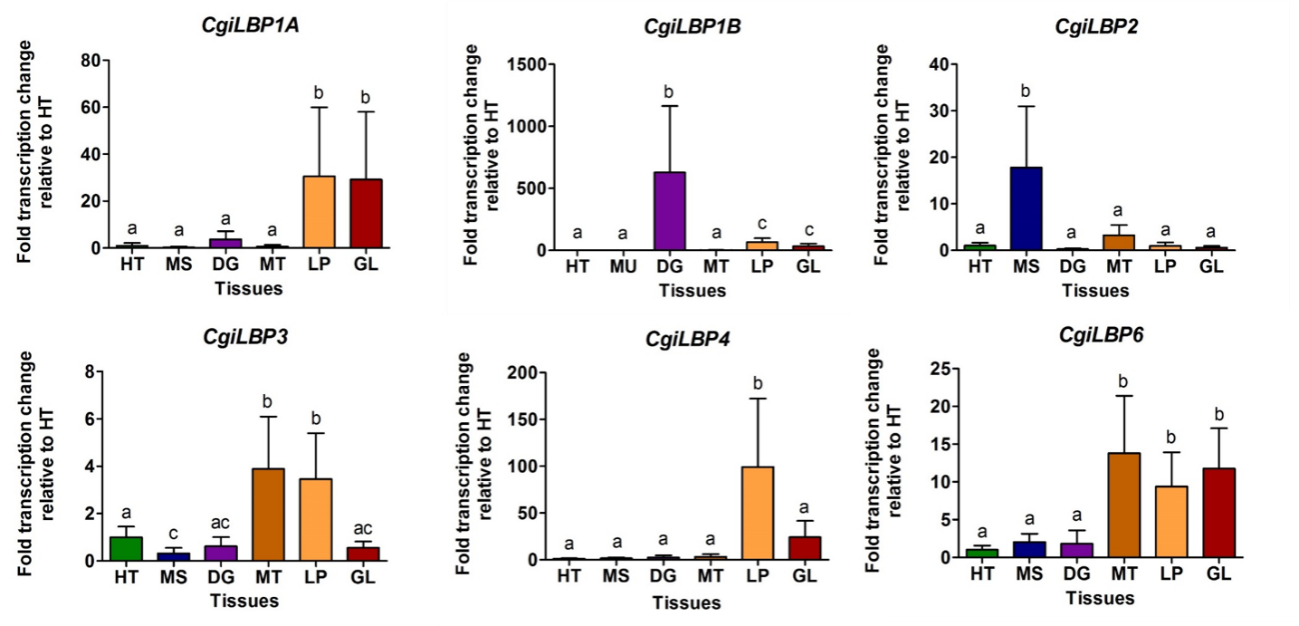

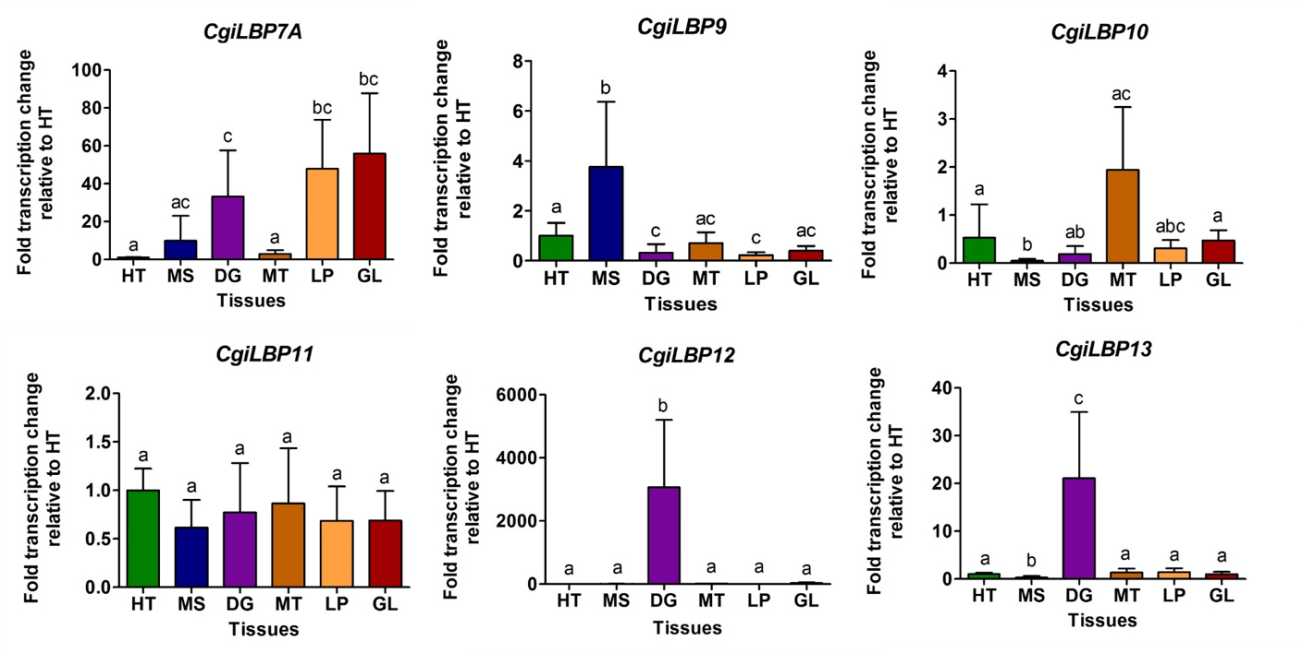


**Figure S6.** Transcript levels of *CgiLBP* genes in different tissues of *Crassostrea gigas*: heart (HT), adductor muscle (MS), digestive gland (DG), mantle (MT), labial palps (LP) and gills (GL). Values are represented as mean fold increase related to HT group ± standard deviation (*p*<0.05).
